# "Conflicted" Conceptions of Conflict of Interest: How the Commercial Sector Responses to the WHO Tool on Conflict of Interest in Nutrition Policy Are Part of Their Standard Playbook to Undermine Public Health Comment on "Towards Preventing and Managing Conflict of Interest in Nutrition Policy? An Analysis of Submissions to a Consultation on a Draft WHO Tool"

**DOI:** 10.34172/ijhpm.2020.164

**Published:** 2020-08-26

**Authors:** A. Rob Moodie

**Affiliations:** Melbourne School of Population and Global Health, University of Melbourne, Melbourne, VIC, Australia.

**Keywords:** Nutrition, Health Governance, Conflict of Interest, Corporate Playbook, Commercial Sector

## Abstract

Managing conflict of interest (CoI) among the interested stake-holders in nutrition policy is a vexed and controversial issue. This commentary builds on Ralston and colleagues’ highly informative analysis of the 44 submissions to the World Health Organization (WHO) draft tool on preventing and managing CoI in national nutrition programs. The commentary proposes that the commercial sector actors are, by definition, too conflicted to objectively respond to the draft tool. The responses of the commercial sectors are predictable, as they mimic their positions during the prior negotiation for the development of the Framework for Engagement of Non-State Actors (FENSA). Their overall approach, and specific responses, are typical of the now standard methods of the ultra-processed food and beverage industry’s ‘corporate playbook.’ In addition, Ralston et al’s analysis raises a number of other issues, such as: why these corporations are so keen to be included in the world of multi-stakeholder partnerships, why so few member states responded to the draft tool, and problems with the term ‘private sector.’ The commentary ends with a suggestion for WHO to seek broader involvement from the 160+ member states who have yet to participate in the consultations regarding the draft tool.

## Introduction

In the last three decades, the global burden of disease due to poor nutrition has increased^1,2^ alongside the rapidly expanding global reach of ultra-processed food and beverage corporations.^3^ These corporations have been otherwise described as the vectors of non-communicable disease epidemics.^4,5^ Despite this, these corporations have become increasingly involved in multi-stakeholder partnerships in global health,^6^ encouraged, for example, by the development of the Sustainable Development Goals.^7^ It has been estimated that trillions of dollars in funding will be required to reach these with the hope that much of it can be sourced from the private sector.^8,9^

It is therefore of little surprise that with the advent of multi-stakeholder, public–private partnerships, in addition to the increasing interest in corporate social responsibility and voluntary pledges by commercial actors, the concept of conflict of interest (CoI) has become one of the most contested issues in contemporary health governance.^10,11^

In their analysis of responses to a draft World Health Organization (WHO) tool for the prevention and management of conflicts of interest.^12^ Ralston and colleagues^13^ have identified the ‘centrality of competing conceptions of CoI’ as a critical challenge to global health governance.

This commentary on their highly informative analysis will focus on the responses of the commercial sector actors^[1]^, how their responses mimic their positions during the Framework for Engagement with non-State actors (FENSA) negotiations, and how their responses form part of the ultra-processed food and beverage industries ‘corporate playbook.’ A number of fascinating issues will be explored such as why these corporations are so keen to be included in the world of multi-stakeholder partnerships; who responded and who did not, and problems with the term ‘private sector.’ The commentary finishes with a suggestion for WHO to continue to seek active involvement from the 160+ member states who have yet to participate in the consultations to date regarding the draft tool.

## Are the Submissions to the Draft WHO Tool to Be Expected?

Before this question is answered, there is another that must be posed. Given that commercial sector actors will potentially be financially impacted depending on how ‘CoI’ is conceptualised, are not they, by definition, too conflicted to contribute objectively to the development of the draft WHO tool? If we believe economist Milton Friedman’s famous dictum “*there is one and only one social responsibility of business--to use its resources and engage in activities designed to increase its profits,*”^14^ then these commercial sector actors must necessarily be in conflict with any policy, regulation, legislation and even any scientific evidence that might have a negative impact on their profits.

The analysis of the 44 submissions highlight dramatically divergent understandings of CoI, with the commercial sector actors adopting an *individual *definition of CoI while the majority of member states, and the non-governmental organizations (NGOs) and academics have adopted an *institutional *definition of CoI. The authors describe policy frames characterised as “collaboration and partnership” by commercial sector actors and “conflicted and restricted engagement” by most member states, NGOs and academics.

The paper notes that “the extent to which respondents supported the tool was closely linked to how they conceptualised CoI.”^13^ Four of the six member states (Brazil, Colombia, Canada, and Namibia) that submitted and the overwhelming majority of academic institutions and NGOs saw the tool as “a potentially important step in strengthening nutrition governance by addressing CoI.”^13^ There were few United Nations (UN) agencies that submitted and it was surprising that the United Nations Children’s Fund, the Food and Agriculture Organization (FAO) and the World Trade Organization (WTO) did not submit given the importance of nutrition and commerce to their mandates. Three NGOs felt that the tool did not go far enough in addressing CoI especially as it related to multi-stakeholder partnerships.

 The commercial sector actors, on the other hand were highly critical of the WHO tool, describing it as “inappropriate, unworkable and incompatible,” revealing “categorical and unhelpful distrust of any private sector” and as “denigrating industry.” These actors counter this perceived bias by framing a collaborative and partnership approach, demonstrating particular adeptness in using these frames to promote policies that are aligned with their economic and political interests.

This framing was to be expected, as these commercial sector actors actively oppose virtually any forms of regulation and have demonstrated a willingness to take this strategy to extreme lengths, in many cases denying the science that underpins the arguments for regulation.^15^ The responses of the commercial actors (and the US Government) to the WHO draft tool are also predictable given their positions during negotiations for the development of FENSA. FENSA^16^ was developed as part of the WHO 2011 reform agenda arising from concerns about how WHO should engage with non-state actors, with particular relevance to the private sector.^17^ A key aspect of FENSA’s considerations is the issue of CoI. The draft WHO tool is different in that it related to CoI from the members states’ perspective but the substantive issues it deals with are very similar to those dealt with by FENSA. One of the reasons for delays in FENSA negotiations was attributed to tensions and disagreements relating to CoI.^18^

The commercial sector actors were very active in lobbying during the FENSA negotiations against what they described as “discriminatory treatment,” arguing for “pro-partnership” framework language which is based on their idiosyncratic conceptualisation of CoI.^18^ This language is again evident in their response to the WHO draft tool.

US business interests had a major influence on FENSA’s negotiations, as they did on the US Government’s position in those negotiations, so it is again little surprise to read the US Government’s negative response to the WHO CoI tool.^18^ Their response beginning with the phrase “we are deeply concerned,”^19^ shows just how much influence that these commercial actors have on the US Government.

The responses of these commercial actors to the draft WHO tool to date do not provide any evidence to dispel the notion described many times before that “that unhealthy commodity industries should have no role in the formation of national or international NCD policy.”^20^

## It Is All Part of Their Playbook

The response of the commercial sector actors to the WHO tool is part of a playbook used for many years to undermine public health.^15,21-23^ It fits neatly into a broader strategy of relentless lobbying directed at national governments and multilateral agencies such as WHO, FAO, and WTO.

And if we return to Milton Friedman, the second half of his famous quote is “…*to increase its profits so long as it stays within the rules of the game, which is to say, engages in open and free competition without deception or fraud*.” Unfortunately, deception has been shown to be a key play in the playbook over many years.^24^

In their responses to the tool, they use the playbook tactic of shifting focus away from themselves toward conflicts of interest amongst other non-state actors. For example, they state the problem is extensive and complex, introducing the notion of ‘white hat’ bias of academics while using academics funded by the food industry as their source.^25^

 They frame CoI as a legitimate but marginal concern that can be effectively addressed by established disclosure practises. CoI is portrayed as being peripheral and manageable, and not something that need narrow the scope of engagement by commercial actors in nutrition policy.

What is intriguing, both in the FENSA negotiations and the responses to the WHO tool, is how much these commercial sector actors resent the fact that they have been constantly been compared to the tobacco industry for so many years. As long as 11 years ago Brownell and Warner have described “the script of the food industry as both similar to and different from the tobacco industry script,” warning that “the world cannot afford a repeat of the tobacco history, in which industry talks about the moral high ground but does not occupy it.”^26^

## Who Responded and Who Did not Respond?

Unfortunately, the time available to respond to the draft tool was only 18 days (from 18-29 September 2017). Of the 44 who provided written feedback, *only six of the 194 member states responded*. The commercial sector provided 14, NGOs provided 12, academic institutions eight and UN/inter-governmental organizations four.

 The short time period worked against the participation of less well-resourced nations and organisations, so it is not surprising that the commercial sector could provide considerably more responses than the member states – and yet the member states are key to the use of the tool.

The only government that responded negatively to the WHO draft tool was the US Government. Interestingly, the FENSA negotiations and the drafting of the WHO tool have occurred at time when the US has a Supreme Court judiciary which is more sympathetic to business interests than any Supreme Court since World War II.^27^ And recent decisions indicate that the justices will continue to restrict the government’s ability to regulate corporate America.^28^ Given the impact that US business culture has on its government’s attitude to the FENSA negotiations^18^ and the influence that the US Government has had on other governments and on multilateral institutions such as WHO, it was no surprise that their “deep concern” cited above about the draft tool, was about the “…overarching tone of exclusion [of the commercial actors]….”^19^

## Why Are the Commercial Sector Actors So Keen to be Associated the WHO and With the UN?

As explained above, the responses from the commercial sector actors are part of an attempt to avoid any form of regulation, motivated by a broader strategy to protect profits. At the same time, their responses highlight a desire to associate with the WHO and the UN, which is an element of their brand management strategy. The more multilateral organisations or national governmental organisations they can be seen to be working with, the greater the “health halo.” It is the global version of having a McDonalds franchise operating in a Children’s Hospital.^29^ At the same time, the closer they are associated, the more they can influence national and international policies – just as we are seeing with their vehement opposition to the draft WHO tool.

The commercial actors have become expert in discourse capture, and they demonstrate this by constantly co-opting the language of the Sustainable Development Goals (SDGs). For example, SDG #17 says “there was a need to increase the space for civil society’s contribution to sustainable development and for* a more inclusive and relevant dialogue between the public and private sectors.”* In the 44 responses to the draft tool, 85% of the total number of references to the SDG’s came from commercial sector actors.

## Why Does Public Health Always Refer to These Particular Commercial Sector Actors as the (Homogenous) Private Sector?

For years, the public health sector has unfortunately been referring to any or all for-profit enterprises, no matter their size, intent, products or processes, as the ‘private sector.’ This problem of categorizing the vast array of different entities under the same term is alluded to in the Ralston et al paper where they refer to “*the need to better differentiate between actors within the food industry, an unhelpfully sweeping category that groups together such diverse entities as community based farming cooperatives and multinational companies.”*^13^

 The commercial actors use this unnuanced public health language to their advantage by repeatedly invoking themselves as part of a much bigger group – the private sector. This enables them to counter their opponents by implying that ‘if you’re against any of us, you must be against all of the private sector.’

 There is now a pressing need for the development of a detailed typology of the huge array of for-profit entities to enable global, national and local health leaders to know with whom it is appropriate to interact, and how this interaction should proceed.

## Is the Tool of Value?

Despite the expected objections from all of the commercial sector actors and the US Government, the authors are correct to claim “*that the tool can offer the potential to move past the blanket acceptance or rejection of partnership to identify specific actors in forms of engagement where conflicts of interest can be managed in ways to protect public health nutrition goals.”*

 They say the tool may offer a useful reference point for member states, officials and policy-makers who are concerned to prevent pursuing collaborations they view as inconsistent with nutrition goals.

 The authors suggest some very sensible amendments to the tool, for example the development of a simplified version as an initial scoping device, clarification of criteria for exclusion and participation, more specific guidance for managing CoI in the context of partnerships and wider technical assistance to enhance governmental capacity.

As a follow up to the consultation in 2017, WHO conducted an “Informal Technical Member States Consultation” in February 2019 which included 21 member states (five of whom were involved in the initial consultation).^30^ This consultation endorsed the “conflicted and restricted engagement” conceptualisation to a much greater degree than the “collaboration and partnership” approach. It covered areas such as practical methods for excluding influence of the private sector in developing dietary guidelines; experts’ declarations of interests, ongoing challenges in managing CoI, use of shorter screening tools; key elements for successful engagement; and the need to strengthen members states capacities to manage CoI.^30^ The WHO secretariat is currently refining the tool.

## A Suggestion

WHO is to be congratulated for developing the tool and conducting the consultations. Given the imperative of avoiding CoI a major push to allow as many member states and national NGOs to participate as possible in utilising the tool is highly desirable, as relatively few were able to respond to the initial consultations. This can be done through regional consultations to encourage the participation of the less well-resourced governments and local NGOs who are often intimidated by the powerful and highly influential supranational food and beverage corporations and their lobbyists.^31,32^

## Ethical issues

 Not applicable.

## Competing interests

 Author declares that he has no competing interests.

## Author’s contribution

 ARM is the single author of the paper.

## Endnotes

 [1] This commentary is focussed on the role of ultra-processed food and beverage industries. The term commercial sector, like the term private sector (see below) is far too comprehensive in this instance. The concern of this commentary is only with those commercial sector actors who are producing, promoting and distributing ultra-processed foods and beverages.
